# The Textural Properties of Extra Virgin Olive Oil (EVOO)-Hydrocolloid Beads and the Quality Parameters of Bosana EVOO as a Preservation Liquid During Bead Shelf Life

**DOI:** 10.3390/foods14091472

**Published:** 2025-04-23

**Authors:** Maria Grazia Farbo, Elisabetta Avitabile, Costantino Fadda, Roberto Cabizza

**Affiliations:** Dipartimento di Agraria, Università degli Studi di Sassari, Viale Italia 39, 07100 Sassari, Italy; mgfarbo@uniss.it (M.G.F.); elybetta@gmail.com (E.A.); cfadda@uniss.it (C.F.)

**Keywords:** beads, Bosana, edible coating, EVOO, hydrocolloids, pectin, shelf life, xanthan gum

## Abstract

The use of edible food packaging by hydrocolloid encapsulation has gained interest as an approach to preserve the physicochemical and sensory properties of food. In this study, pectin (PE) and xanthan gum (XG), naturally occurring hydrocolloids, were utilized with calcium chloride (CaCl_2_) as a bead-forming agent to develop an extra virgin olive oil-hydrocolloid emulsion encapsulating Bosana extra virgin olive oil (EVOO), a Sardinian monovarietal oil rich in polyphenols and sensory properties. This study investigated the textural evolution of EVOO-beads immersed in Bosana EVOO as a preservative liquid (PL) during 180 days of storage at 20 °C (room temperature) and 40 °C (accelerated shelf-life test). The bead texture was assessed at 30-day intervals along with selected oil quality parameters. Its hardness remained stable, while its springiness, cohesiveness and chewiness significantly decreased with time. Temperature and the interaction time x temperature were significant for cohesiveness. PL showed the expected degradation of polyphenols and α-tocopherol influenced by storage time and temperature. At 20 °C, free acidity and peroxide levels remained within EVOO quality standards, confirming the protective role of encapsulation. Between the PL and oil controls, no effect of the beads was observed. These results highlight the potential of hydrocolloid-based encapsulation to produce EVOO-beads, offering innovative applications as functional food coatings and in preservation technologies.

## 1. Introduction

In recent years, there has been a notable advancement in the development of encapsulation systems for vegetable oils, with the aim of creating functional or innovative products [1,2,3]. Extra virgin olive oil (EVOO) is one of the best-known vegetable oils, mainly produced in the Mediterranean area for millennia, and it is fundamental to the Mediterranean diet [4,5]. EVOO is obtained from the fruit of the *Olea europaea* L. tree, with a unique composition and properties [6,7,8,9]. EVOO is among the few oils that can be consumed in its natural form, retaining all its natural constituents [10,11]. The high polyphenol content in extra virgin olive oil reduces inflammation, obesity, cancer incidence, and cardiovascular disease risk and mortality in high-risk individuals by lowering blood pressure, while its phenolic compounds protect against cognitive and neurological deficits through their free radical scavenging activity [12,13,14,15,16]. Phenolic compounds have a protective action against cognitive and neurological deficits thanks to the scavenging activity of the high concentrations of free radicals. The presence of phenolic compounds influences the shelf life of olive oil [17,18,19]. Bosana is the most prevalent cultivar in Sardinia and one of the most important in Italy [20]. The population of this variety is estimated to be approximately three million plants, predominantly concentrated in the northwestern area of the island. Bosana EVOO is characterized by its polyphenol content and sensory profile [21].

Encapsulation is a technique used to retain active compounds within a matrix in particle form [22,23,24,25]. Numerous studies have documented the encapsulation of various oils for applications in nutrition, therapeutics, aromatics, food, and nutraceuticals [26,27,28,29,30]. Among these, the encapsulation of EVOO in innovative packaging has emerged as a promising approach [29]. The selection of encapsulating matrices and gums—preferably of natural origin—is guided by considerations of application, safety, and cost-effectiveness [31]. Hydrocolloids, known for their versatile texturing properties, play a crucial role in modern food processing [32]. Specifically, pectin (PE) and xanthan gum (XG) are natural hydrocolloids well-suited for encapsulation due to their stabilizing and gel-forming capabilities [33].

PE is a linear polysaccharide containing about 300 to 1000 monosaccharide units. The structure of PE is a polymer of α-D-galacturonic acid with 1 → 4 bonds interrupted by segments of rhamnogalacturonan, which combine residues of galacturonic acid and α-L-rhamnopyranose from a 1 → 2 bond that is a partially esterified methyl [34,35]. PE is extracted from apples or citrus peels and is used as a food additive and a gelling and thickening agent [36,37]. It can reduce the interfacial tension between an oil phase and an aqueous phase and is efficient in the encapsulation of oil matrices [24].

XG is a natural biopolymer (polysaccharide) composed of β-D-glucose units linked at the 1 and 4 positions, known primarily for its gelling properties [38,39,40]. Due to its non-toxic, biocompatible and biodegradable nature, it is widely used as a stabilizer and emulsifier in various food products and formulations. In the food industry, it plays a crucial role in the production of edible coatings and biodegradable packaging films. In addition, xanthan gum is highly valued as a stabilizer due to its excellent temperature and pH stability, compatibility with various food ingredients and rheological properties [41]. In particular, it maintains optimal stability and viscosity even at high temperatures, making it a versatile ingredient in food applications [42,43,44,45,46,47,48].

The purpose of this study was to develop an edible encapsulation system for EVOO using PE and XG, making it suitable for food industry applications. At the same time, this research assessed the texture profile of EVOO beads over time to evaluate their rheological behaviour. Furthermore, an investigation was conducted into the quality parameters of the preserving liquid during its shelf life. In addition, the choice of Bosana EVOO as a medium for storing the beads was based on its recognized nutritional and sensory qualities, and its role in the creation of a territorial identity as part of the formulation.

The present study specifically investigated the influence of storage time and temperature on the product. This approach aimed to provide insights into the stability and potential industrial applications of PE-XG-EVOO beads stored in EVOO.

## 2. Materials and Methods

### 2.1. Chemicals and Reagents

Methanol (MeOH), hexane, 2-propanol, sodium carbonate and Folin–Ciocalteu reagent were supplied by Carlo Erba (Milano, Italy). Pectin, and ClCa_2_ were purchased from Sigma–Aldrich (St. Louis, MI, USA). Xanthan gum powder was obtained from Chimab (Campodarsego, Italy).

### 2.2. Extra Virgin Olive Oil

EVOO was kindly provided by Accademia Olearia srl (Alghero, Italy) and obtained from the Bosana olive cultivar using a LEOPARD multiphase decanter (DMF) (Pieralisi, Jesi, Italy), during the 2021–2022 harvesting season as previously described in Dahdah et al. [49,50]. Briefly, Bosana mono cultivar EVOO was obtained using the Leopard DMF, a two-phase centrifuge able to produce a little hydrated pomace, like that obtained from a three-phase decanter, and recovering a certain amount of pulp from the pomace to generate an olive pomace, without adding water to the olive paste during the malaxation step. The Bosana EVOO is stored in dark glass bottles at room temperature in the dark until it is used.

### 2.3. Preparation of EVOO beads

Pectin (PE) solution (2% *w*/*v*) was prepared in reverse osmosis water. XG powder (1% *w*/*v*) was dispersed in reverse osmosis water, heated to 55 °C, and cooled to room temperature to create a homogenous, transparent, and stable gel.

**Figure 1:**
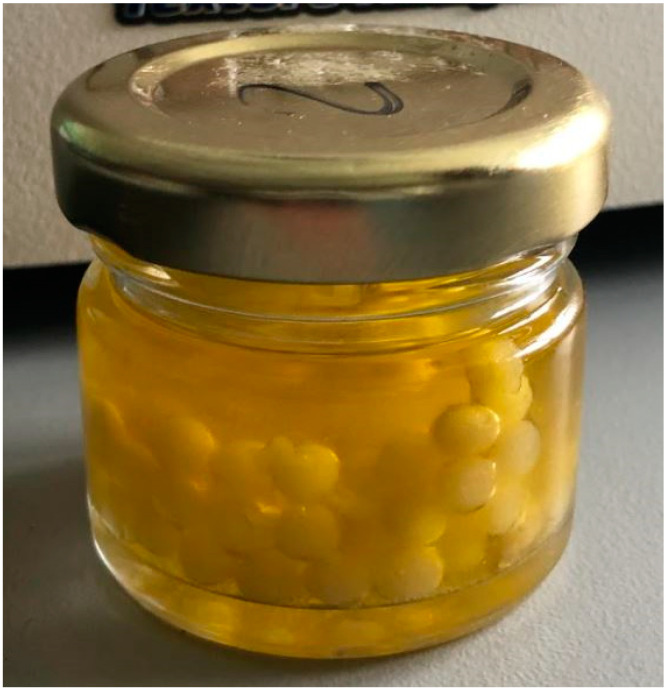
A glass jar containing EVOO beads dispersed into Bosana EVOO as a preserving liquid (PL).

An oil–hydrocolloid emulsion was prepared by adding 0.5 mL of XG (sol. 1%), 2 mL of PE (sol. 2%) and 1.5 mL of EVOO. The formulation was mixed by vortex for 1 min (ZX3 Velp Scientifica, Usmate, Italy), transferred in a 5 mL syringe without a needle (Ing Light Leur Lock, Rays, Milan, Italy), allowing drop formation by gravity, and then dropped into the solution of ClCa_2_ 1% (*w*/*v*) under conditions of gentle stirring for two minutes. Subsequently, the beads were rinsed with reverse osmosis water. Finally, fifty beads with a diameter of approximately 5 mm, filled with 1.5 mL of Bosana EVOO, were placed in glass jars. Afterwards, the jars were filled with 15 mL of the same EVOO to act as a preservation liquid and then closed (Figure 1).

**Figure 2:**
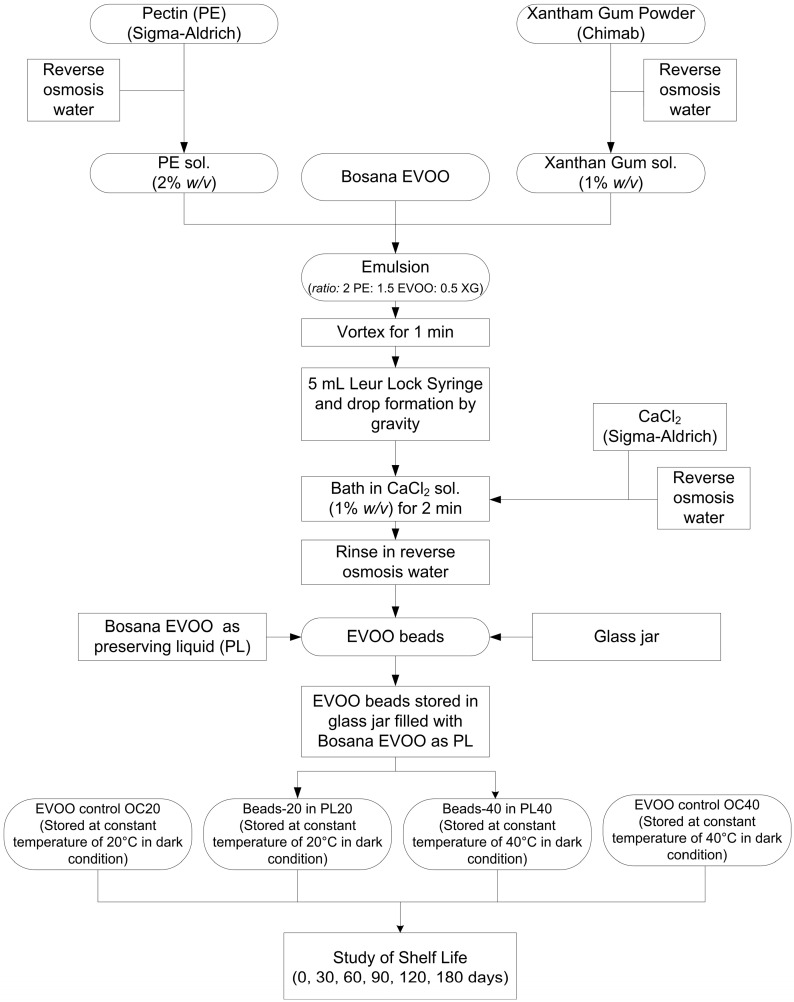
Flow chart of EVOO bead production and shelf life study.

The glass jars were stored in the dark condition in two different laboratory ovens at constant temperatures of 20 °C (beads-20, and PL20) or 40 °C (beads-40, and PL40) (UN160, Memmert GmbH + Co. KG, Schwabach, Germany) for 6 months and sampled every 30 days (0, 30, 60, 90, 120, 150, and 180 days) (Figure 2). Two control samples of Bosana EVOO were prepared and stored at 20 °C (OC20) or 40 °C (OC40) under the same conditions as the oil containing the beads. These control oils were then analysed for quality parameters at the same time points as the preserving liquids in order to assess any potential influence of the beads on the evolution of oil stability over time. There were two replicates for each treatment.

### 2.4. Texture Profile Analysis (TPA) of EVOO beads

The texture profile analysis of EVOO beads was performed using a TA-XT2 plus Texture Analyser (Stable Micro Systems Ltd., Godalming, UK) fitted with a 30 kg load cell. Texture Exponent Software TEE32, version 6.1.10.0 (Stable Micro System, Surrey, UK), was used for data processing. The TPA test was carried out on 15 beads from each oven (beads-20 and beads-40) at each time point of storage. A compression plate (100 mm diameter) was used to compress the samples at a constant speed of 1 mm/s with a 30 s interval between compressions. The force necessary to compress the samples by 50% of their original width was determined from the force–time curve. From the resulting force–time curves, the parameters of hardness, springiness, cohesiveness, and chewiness, were investigated. All measurements were taken in duplicate for each condition.

### 2.5. Free Acidity and Peroxide Value of Preserving Oil

Free acidity and peroxide value were chosen for the oil quality parameters. Free acidity and peroxide value in oil were analysed according to EEC/2568/91 [51]. Free acidity was expressed as % of oleic acid, and peroxide value was defined as m_eq_ of active O_2_/kg of oil. All measurements were taken in duplicate for each condition.

### 2.6. Total Phenolic Content (TPC) of Preserving Oil

Phenolic compounds were extracted from the oil samples according to Conte et al. [5] with some modifications, and total phenolic content (TPC) was measured. In detail, 5 g of oil was mixed with 2 mL of hexane and 2 mL of MeOH:H_2_O 70:30 (*v*/*v*), then centrifuged at 6000 rpm at 4 °C for 10 min (Neya 16 R, Remi Elektrotechnki Ltd., Vasai, India). The hydroalcoholic phase was then collected and further centrifuged at 9000 rpm for 5 min at room temperature, and the resulting extract was filtered through a nylon filter (0.45 mm, VWR, Milan, Italy). Subsequently, 0.5 mL of the sample was mixed with 2.5 mL of Folin–Ciocalteu reagent [52], and vortexed. After 3 min, 5 mL of sodium carbonate (20%) was added, mixed and made up to 50 mL with reverse osmosis water. The extracts obtained were incubated for 90 min at room temperature in the dark and analysed spectrophotometrically at 760 nm (Cary 3500 UV-Vis, Agilent Technologies, Santa Clara, CA, USA). The results are expressed in mg Gallic acid equivalent (GAE) per kg of oil (mg GAE/kg), and each sample was measured twice at each time point of the storage time course. All measurements were taken in duplicate for each condition.

### 2.7. α-Tocopherol Determination by HPLC of Preserving Oil

The investigation and quantification of α-tocopherol (as mg/kg) was conducted using the method and equipment employed in the research of Conte et al. [5]. The equipment consisted of an HPLC Agilent 1100 Series (Agilent Technologies, Palo Alto, CA, USA) coupled with a detector FLD Agilent 1100 Series set at 290 nm for excitation and 330 nm for emission and a C18 Gemini Column 100 × 4.6 mm ID, 3 mm particle size column (Phenomenex, Torrence, CA, USA). The mobile phase used was MeOH:H_2_O 96:4 (*v*/*v*) with a flow rate of 2 mL/min [53]. All measurements were taken in duplicate for each condition.

### 2.8. Statistical Analysis

Obtained data were analysed by a two-way analysis of variance (ANOVA) using Statgraphics Centurion XVI for Windows software package (version 16.2.04; Statpoint Technologies, Inc. Warrenton, Virginia, VA, USA). Two-way ANOVA was performed to evaluate the effect of temperature (20 °C, and 40 °C), day (0, 30, 60, 90, 120, 150, and 180) and their interaction on the structure of the beads and the selected quality parameters of the PL. When significant differences were found (*p*-value < 0.05), Fisher’s Least Significant Difference (LSD) test was applied as a post hoc analysis to determine pairwise differences between means.

Furthermore, a Student’s *t*-test was employed to compare the acidity, peroxide, TPC and α-tocopherol values between PL and OC at each time point. The tests were conducted independently at each storage interval for both the 20 °C and 40 °C conditions. Statistical significance was considered as *p* < 0.05.

## 3. Results

### 3.1. Texture Profile Analysis of beads

**Figure 3:**
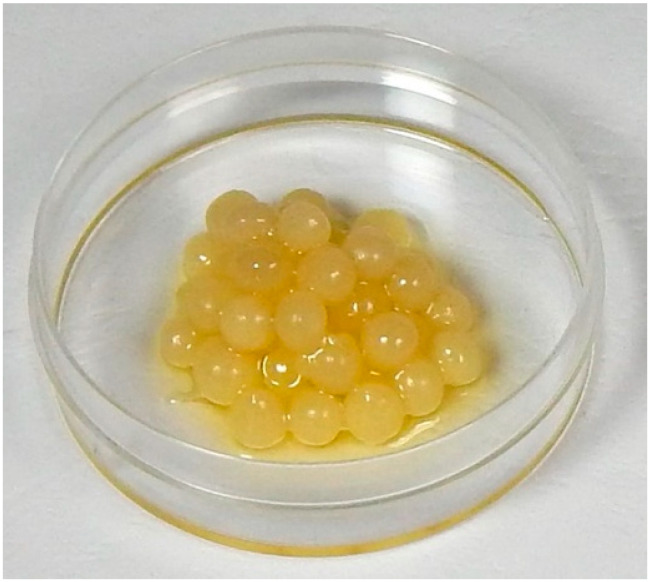
EVOO-beads in a Petri dish.

The emulsion of Bosana EVOO and natural hydrocolloids resulted in the formation of spherical beads (Figure 3).

The oil was encapsulated within a polymeric network with strong hydrocolloid–hydrocolloid interactions, stabilized by the addition of XG. According to the literature, XG may contribute to increasing the viscosity of the continuous phase, potentially helping to prevent oil coalescence [54,55]. The interaction of XG with PE could also increase the thickness of the surface layer of the beads and improve its mechanical strength [56]. The results of the TPA compression analysis are reported in Table 1. The hardness (N), defined as the peak force that occurs during the initial compression, of EVOO beads was not affected by time (*p* > 0.05), temperature (*p* > 0.05), or their interaction (*p* > 0.05). This indicates that the hardness remained stable regardless of the storage duration or the storage temperature.

Springiness (S) is defined as the height that the food recovers during the time that elapses between the end of the first bite and the start of the second bite. It is a measure of the rate at which a deformed material reverts to its undeformed condition after the removal of the deformed force [57]. This parameter is associated with the concentration and presence of hydrocolloids and oil incorporated in the beads.

It was observed that the effect of time had a strong impact on the bead’s springiness (*p* < 0.05). On the other hand, no effect of temperature (*p* > 0.05) or even time–temperature interaction (*p* > 0.05) was observed on the springiness of the beads. The stronger effect of time on the beads’ springiness could be attributable to a combination of structural and physical–chemical changes, which occur over time. One of the possible effects may be a reorganization of the polysaccharide network with rearrangements that modify the springiness of the beads [58]. In addition, the interaction of the emulsion of PE-XG and EVOO compounds may have an effect on the viscoelastic properties of the beads, with a consequent reduction in springiness.

Cohesiveness is a parameter used to indicate the strength of the internal bonds of food [57]. As reported in Table 1, it was revealed that there was a significant effect of factor time (*p* < 0.05), suggesting structural modification of the gel matrix. Also, the temperature was observed to have a significant effect (*p* < 0.05), indicating that a higher temperature modifies the cohesiveness. Higher temperatures may lead to a reduction in the interactions between hydrocolloids and EVOO, affecting the structure of the beads [59]. Finally, the interaction between time and temperature was significant (*p* < 0.05), suggesting that cohesiveness does not undergo uniform modification across varying storage conditions. High temperatures could accelerate the structural modification occurring over time.

Chewiness (N) represents the energy needed to chew food, and it is an important consistency characteristic of EVOO beads. The results of this study demonstrated a statistically significant effect of time on the chewiness of the beads (*p* < 0.05), while temperature did not exhibit a significant effect (*p* > 0.05). Most likely, during storage, a reorganization of the hydrocolloid network occurs that modifies the mechanical resistance of the gel matrix. Furthermore, the interaction between time and temperature proved to be non-significant (*p* > 0.05), indicating that the effect of time on chewiness occurs independently of the storage temperature.

The beads demonstrated consistent hardness, suggesting that their structural integrity remains intact despite alterations in other textural properties. The parameters of cohesiveness, springiness, and chewiness exhibited a reduction over the temporal progression, with cohesiveness being additionally influenced by temperature. Temperature control is key for preserving the structure and texture of the beads over time, as higher temperatures increase the loss of cohesiveness.

To date, there have been no studies comparing the texture of beads produced under similar conditions, either using the same hydrocolloids or immersed in the control liquid for the shelf-life study.

### 3.2. Determination of Acidity and Peroxide Values of Preserving Liquid

The free acidity of the oils was strongly affected by the storage time (*p* < 0.05), as reported in Table 2. The samples showed a constant increase in free acidity during the time course. This result was expected due to the hydrolytic and oxidative degradation process that occurs in EVOO over time [60,61]. The effect of temperature was also significant (*p* > 0.05), indicating that high temperatures accelerate oxidative and hydrolytic reactions, leading to faster deterioration of the oil quality [62]. In fact, it reinforces the importance of storage temperature in the maintenance of low acidity and the prevention of oil degradation. The interaction between time and temperature is also statistically significant (*p* < 0.05). The samples stored under the conditions of the ASLT exceeded the basic requirement for extra virgin olive oil fixed at an acidity of 0.8% after 180 days of storage (PL40; 0.86% of oleic acid). In contrast, PL20 exhibited an acidity level of 0.75% at the end of the storage period of 180 days, indicating that the Bosana olive oil employed in the experimental study retained its extra virgin designation at the conclusion of the study. At both 20 °C and 40 °C, no statistically significant differences in acidity were observed between the oil samples containing EVOO beads and the respective EVOO controls (OC20 and OC40) at any observation time point (*p* > 0.05). These findings suggest that the presence of the beads did not affect the hydrolytic degradation of the oil during the storage period under either condition.

The acidity of the preserving liquid exhibited first-order kinetics in both samples during storage (PL20 R^2^ = 0.95; PL-40 R^2^ = 0.98). The equations that were deduced from these data were as follows: for the acidity of the PL20 sample, ln(C) = 0.0050t − 1.2958; and for the PL40 sample, ln(C)= −0.0057t − 1.1822. A comparison of the data indicates, as expected, the k (rate constant) was higher in samples stored at a temperature of 40 °C, suggesting an increase in the degradation process of extra virgin olive oil. The calculated activation energy (E_a_) for the increase in acidity was 5.00 kJ/mol, indicating that the hydrolysis and oxidation process responsible for the rise in free fatty acids occurs with minimal energy input and is significantly accelerated by temperature. This highlights the importance of proper storage conditions to slow down acidification and preserve the quality of EVOO over time.

A significant effect of storage time on the peroxide value of the preservation fluids (*p* < 0.05) was observed (Table 2). These results are consistent with previous studies that reported an increase in peroxide with storage time caused by the progressive oxidation of unsaturated fatty acids present in EVOO [60,61]. Also, temperature showed a significant effect on the increase of peroxide values (*p* < 0.05), especially 40 °C, which led to higher values compared to 20 °C. High temperatures enhance lipid peroxidation, causing a faster breaking down of fatty acids and forming primary oxidation products [62]. The interaction between storage time and temperature was statistically significant (*p* < 0.05), confirming that the increase in the peroxide value depends on both factors.

After 180 days, the peroxide value of PL20 remained below the threshold of 20 m_eq_ O_2_/kg (19.65 ± 0.21 m_eq_ O_2_/kg), whereas PL40, after 90 days of storage, exceeded the peroxide limit for extra virgin olive oil, reaching 22.52 m_eq_ O_2_/kg, causing a downgrade as required by the regulations. Statistical analyses were conducted on the data, revealing no significant differences in peroxide values between the preserving liquids and the corresponding controls at any time point (*p* > 0.05), under both 20 °C and 40 °C storage conditions. This finding indicates that the presence of the EVOO-beads did not affect the oxidative stability of the Bosana oil during the storage period.

The trend of peroxide evolution fitted a quadratic regression in both samples for the observed period. For the PL20 samples, the given equation was as follows: y = 0.00009x^2^ + 0.0366x + 9.8569 (R^2^ = 0.98), and for the PL40 samples, the equation was: y= −0.0005x^2^ + 0.1732x + 10.139 (R^2^ = 0.99). The quadratic term of PL40 indicates that the initial reaction is faster but then it tends to stabilize or slow down. This indicates possible saturation due to the decomposition of peroxides into oxidation byproducts after a certain point. The linear coefficient of PL40 was higher than PL20 (0.1732 vs. 0.0366), indicating a faster oxidation caused by a higher temperature. At 20 °C, the increase is more gradual and constant over time, with no clear indication of stabilization over the observed period.

### 3.3. Determination of Total Phenolic Content of Preserving Liquid

The TPC in Bosana EVOO oil was 247.2 mg of Gallic acid equivalent per kg at day zero. The findings of this study demonstrated that storage duration exerts a highly significant effect on TPC (*p* < 0.05), indicating a substantial decrease over time (Table 2). This is consistent with the results of previous studies, which have shown that polyphenols undergo oxidative and hydrolytic degradation during prolonged storage [63]. Temperature significantly affected the TPC levels (*p* < 0.05), with a more pronounced decrease at 40 °C compared to 20 °C. This is consistent with research showing that elevated storage temperatures accelerate polyphenol oxidation and enzymatic degradation, reducing antioxidant activity [63,64].

The significant interaction between time and temperature (*p* < 0.05) suggests that the rate of polyphenol degradation depends on the storage temperature, with a faster decline at 40 °C than at 20 °C. This confirms the findings of studies reporting that polyphenol retention is temperature dependent, with higher temperatures leading to greater oxidative losses [61,64].

A 31.4% decrease in TPC was observed at the end of the shelf life studied in preserving liquid at 20 °C, while a 40.7% reduction in TPC was recorded at 40 °C.

There were no statistically significant differences in total phenolic content at 20 °C or 40 °C between oil samples containing EVOO beads (PL20 and PL40) and the controls (OC20 and OC40) at any time point (*p* > 0.05). The presence of the beads did not affect the phenolic stability of the oil.

The degradation rate of polyphenols followed in both samples was a quadratic regression. The equations of samples were as follows: PL20 y = 0.0023x^2^ − 0.8443x + 249.74 (R^2^ = 0.99); PL40 y = 0.0033x^2^ − 1.0933x + 243.98 (R^2^ = 0.93). Comparing the models, the rate of polyphenol degradation was higher when the preserving liquid was stored at 40 °C compared to 20 °C. Moreover, the initial loss of content in polyphenols was higher in PL40, and the degradation curve at 40 °C showed a steeper trend than at 20 °C (Table 2). The degradation acceleration factor (F) was found to be 1.31 times higher in PL-40 than in PL-20. This indicates that degradation occurred at a 31% faster rate when the temperature was doubled from 20 °C to 40 °C.

These results are in accordance with other scientific studies reported in the literature for a decrease in total phenolic content in EVOO samples during storage, although studied under different conditions. In fact, Castillo Luna [65] monitored the phenolic decrease in 160 Spanish EVOOs after 12 months of storage in darkness at 20 °C, noting a reduction in the content of total phenolic compounds of 42 ± 24.3%. This reduction was found to be significantly correlated with the initial phenolic profile, including oleacein and oleocanthal, which the author associated with a notable decrease in total phenolic content. Conversely, hydroxytyrosol and oleocanthalic acid demonstrated a marked increase following storage at 20 °C.

Krichene et al. [64] studied the stability of phenolic compounds on monovarietal EVOO under different conditions (closed CB, and open bottle OB) at different storage temperatures. The stability of phenolic compounds was found to vary according to cultivar and storage temperature. For instance, a decrease of 37% in CB and 48% in OB was observed in Chemlali VOO, a Tunisian cultivar, after six months of storage at 25 °C. In the same study, a reduction of total phenolics of 21% was noted in monovarietal El Hori EVOO after storage for 5.5 months at 25 °C. Mancebo-Campos et al. [66] noted a pseudo first-order kinetic behaviour in total phenols content in samples of Spanish EVOO stored in open glass bottles at different temperatures (25, 40, 50 and 60 °C) for 93, 41, 34 and 19 weeks.

### 3.4. α-Tocopherol Content of Preserving Liquid

In the initial phase of the study (day 0), the concentration of α-tocopherol was recorded to be 243.65 ± 1.90 mg/kg. The behaviour of the α-tocopherol content significantly decreased over time (*p* < 0.05), as reported in Table 2. This result aligns with previous research on the oxidative and hydrolytic degradation of α-tocopherol during storage [67]. Higher storage temperatures (40 °C) significantly accelerated the degradation of α-tocopherols compared to 20 °C. This result is consistent with studies showing that α-tocopherol oxidation and loss are temperature-dependent, with increased degradation at elevated temperatures due to lipid oxidation [66]. The interaction effect indicates that α-tocopherol degradation is more pronounced at 40 °C, suggesting that oxidative mechanisms are intensified at higher temperatures.

Throughout the storage period, no statistically significant differences in α-tocopherol content were observed between samples containing EVOO beads in preserving liquid and control oils at either 20 °C or 40 °C (*p* > 0.05). These findings suggest that the presence of the beads did not influence the oxidative degradation of α-tocopherol under standard or accelerated storage conditions.

α-tocopherol levels followed a quadratic regression in both samples analysed, similar to the degradation of polyphenols during storage. Indeed, both the PL20 and the PL40 were modelled on the following equations: PL20 y = 0.0016x^2^ − 0.652x + 241.74 (R^2^ = 0.95); PL40 y = 0.0023x^2^ − 0.9973x + 241.22 (R^2^ = 0.98). It was observed that, at both temperatures, there was a decrease in α-tocopherol content over time, following a non-linear trend. The rate of degradation was found to be faster at 40 °C. It is evident from the data that the linear coefficient of PL40 is more negative than that of PL20 (−0.9973x vs. −0.652x), which indicates a marked loss of α-tocopherol due to exposure to higher temperatures. The quadratic coefficient was higher for PL40 (0.0023x^2^) than PL20 (0.0016x^2^), suggesting that degradation accelerates more rapidly over time with increasing temperature. This finding indicates that to optimize the preservation of α-tocopherol in products, it is recommended to maintain them at reduced temperatures in order to slow down the effects of increased oxidation or heat-accelerated chemical reactions. Krichene et al. [64] noted a degradation of α-tocopherol, apparently of a zero-order kinetic, with a lag phase of a few months, with the exception of samples stored at 50 °C and those exposed to oxygen, which showed a marked decrease. Mancebo-Campos et al. [66] observed a comparable response in all samples exposed to varying temperatures, exhibiting a pseudo zero-order kinetic. Their experiment noted a reduction in α-tocopherol in a range of 12–28% for the sample stored at 25 °C and 26–56% for the 40 °C sample. This was in accordance with the data recorded in this study, where a reduction of 29.3% was noted in PL20 and 45.8% in PL40. A significant degradation rate may be attributable to the high susceptibility of α-tocopherol to oxidize into α-tocopherol quinones at elevated temperatures [68].

## 4. Conclusions

This study highlights the importance of storage conditions in preserving the textural properties of encapsulated EVOO using hydrocolloids. The findings of this study demonstrated that encapsulation was an effective method of maintaining bead hardness at elevated temperatures and during storage. However, it was also observed that time exerted a negative effect on springiness, cohesiveness and chewiness. Moreover, the interaction time x temperature was significant for cohesiveness. At a temperature of 20 °C, the acidity and peroxide value of PL remained within the legal parameters of EVOO for 180 days. However, in the ASLT condition, the acidity and peroxide value of the oil exceeded the acceptable threshold before the 180-day deadline. The presence of the beads in EVOO, utilised as the preserving liquid, did not exert any noticeable positive or negative effect on selected parameters analysed, in comparison to the control oil stored under the same temperature and time conditions.

This outcome demonstrates the protective effect of lower temperatures on the quality of extra virgin olive oil. In order to minimize the stability of EVOO encapsulated in PE-XG beads, it is recommended to store the product at lower temperatures and in dark conditions to reduce polyphenol and α-tocopherol degradation and maximize the stability of the EVOO. These findings contribute to the development of functional edible coatings and oil preservation strategies, paving the way for new EVOO products in the food industry. Further research should be conducted into the optimization of hydrocolloid formulations, with a particular focus on the sensory component of EVOO beads and its acceptance by consumers. In addition, further research is warranted on the integration of EVOO beads, in their innovative format, into diverse food matrices, including dairy products (fresh cheese, ricotta and yoghurt), as well as bakery goods, and applications in haute cuisine and molecular gastronomy.

## Figures and Tables

**Table 1:** Two-way Analysis of Variance (ANOVA) on Texture Profile Analysis of EVOO beads.

Beads	Hardness (N)	Springiness	Cohesiveness	Chewiness (N)
Temperature				
20 °C	0.014 ± 0.006 ^a^	0.93 ± 0.06 ^a^	0.28 ± 0.02 ^a^	3.71 × 10^−3^ ± 0.002 ^a^
40 °C	0.013 ± 0.005 ^a^	0.86 ± 0.07 ^a^	0.27 ± 0.04 ^b^	3.35 × 10^−3^ ± 0.002 ^a^
*p*-value	0.66	0.22	0.000	0.13
Day				
0	0.015 ± 0.006 ^a^	0.98 ± 0.02 ^a^	0.29 ± 0.01 ^a^	4.47 × 10^−3^ ± 0.002 ^a^
30	0.014 ± 0.006 ^a^	0.94 ± 0.06 ^ab^	0.29 ± 0.03 ^a^	4.25 × 10^−3^ ± 0.002 ^ab^
60	0.014 ± 0.003 ^a^	0.92 ± 0.07 ^bc^	0.28 ± 0.02 ^a^	4.11 × 10^−3^ ± 0.002 ^ab^
90	0.014 ± 0.006 ^a^	0.90 ± 0.06 ^bc^	0.28 ± 0.03 ^a^	3.52 × 10^−3^ ± 0.002 ^bc^
120	0.014 ± 0.005 ^a^	0.90 ± 0.06 ^c^	0.26 ± 0.04 ^b^	3.26 × 10^−3^ ± 0.002 ^c^
150	0.012 ± 0.006 ^a^	0.87 ± 0.08 ^d^	0.26 ± 0.04 ^b^	2.88 × 10^−3^ ± 0.001 ^c^
180	0.012 ± 0.005 ^a^	0.86 ± 0.07 ^d^	0.25 ± 0.04 ^b^	2.74 × 10^−3^ ± 0.001 ^c^
*p*-value	0.17	0.000	0.000	0.000
Temperature × Day				
0 × 20	0.015 ± 0.006 ^a^	1.05 ± 0.02 ^a^	0.29 ± 0.05 ^a^	4.56 × 10^−3^ ± 0.002 ^a^
0 × 40	0.015 ± 0.006 ^a^	1.05 ± 0.02 ^a^	0.29 ± 0.05 ^a^	4.56 × 10^−3^ ± 0.002 ^a^
30 × 20	0.014 ± 0.006 ^a^	1.05 ± 0.14 ^a^	0.29 ± 0.03 ^a^	4.50 × 10^−3^ ± 0.002 ^a^
30 × 40	0.014 ± 0.006 ^a^	1.05 ± 0.13 ^a^	0.28 ± 0.03 ^a^	3.92 × 10^−3^ ± 0.002 ^a^
60 × 20	0.014 ± 0.004 ^a^	1.05 ± 0.10 ^a^	0.28 ± 0.01 ^a^	4.00 × 10^−3^ ± 0.002 ^a^
60 × 40	0.014 ± 0.003 ^a^	1.05 ± 0.10 ^a^	0.28 ± 0.02 ^a^	4.31 × 10^−3^ ± 0.002 ^a^
90 × 20	0.014 ± 0.006 ^a^	0.94 ± 0.11 ^a^	0.28 ± 0.03 ^a^	3.38 × 10^−3^ ± 0.002 ^a^
90 × 40	0.014 ± 0.006 ^a^	0.95 ± 0.03 ^a^	0.28 ± 0.03 ^a^	3.56 × 10^−3^ ± 0.001 ^a^
120 × 20	0.014 ± 0.005 ^a^	0.92 ± 0.08 ^a^	0.28 ± 0.03 ^a^	3.38 × 10^−3^ ± 0.002 ^a^
120 × 40	0.012 ± 0.004 ^a^	0.91 ± 0.07 ^a^	0.24 ± 0.02 ^b^	3.15 × 10^−3^ ± 0.001 ^a^
150 × 20	0.012 ± 0.006 ^a^	0.92 ± 0.07 ^a^	0.28 ± 0.03 ^a^	2.88 × 10^−3^ ± 0.002 ^a^
150 × 40	0.012 ± 0.006 ^a^	0.83 ± 0.07 ^b^	0.23 ± 0.03 ^b^	2.50 × 10^−3^ ± 0.001 ^a^
180 × 20	0.012 ± 0.006 ^a^	0.91 ± 0.08 ^a^	0.28 ± 0.02 ^a^	3.13 × 10^−3^ ± 0.001 ^a^
180 × 40	0.012 ± 0.004 ^a^	0.82 ± 0.04 ^b^	0.23 ± 0.04 ^b^	2.25 × 10^−3^ ± 0.001 ^a^
*p*-value	0.97	0.19	0.000	0.39

Each value represents the mean ± SD. Different superscripts within the same column indicate significant differences as assessed by a two-way ANOVA, followed by Fisher’s Least Significant Difference (LSD) test applied as a post hoc analysis to determine pairwise differences between means.

**Table 2:** Two-way Analysis of Variance (ANOVA) on the selected parameters of preserving liquids.

Preserving Liquids	Free Acidity (% Oleic Acid)	Peroxide Value (m_eq_ O_2_/kg)	TPC (mg GAE/kg)	α-Tocopherol (mg/kg)
Temperature				
20 °C	0.45 ± 0.15 ^b^	14.23± 3.36 ^b^	206.5 ± 31.3 ^a^	204.6 ± 25.2 ^a^
40 °C	0.50 ± 0.18 ^a^	20.06 ± 5.62 ^a^	191.9 ± 38.1 ^b^	182.5 ± 39.8 ^b^
*p*-value	0.000	0.000	0.000	0.000
Day				
0	0.28 ± 0.01 ^g^	9.86± 0.08 ^g^	251.6 ± 1.7 ^a^	243.6 ± 1.7 ^a^
30	0.36 ± 0.04 ^f^	13.27 ± 2.52 ^f^	205.5 ± 15.8 ^b^	219.4 ± 6.5 ^b^
60	0.41 ± 0.04 ^e^	15.07 ± 3.70 ^e^	197.7 ± 14.9 ^c^	187.5 ± 11.8 ^c^
90	0.46 ± 0.07 ^d^	18.56 ± 4.57 ^d^	185.0 ± 7.0 ^d^	184.1 ± 14.4 ^d^
120	0.54 ± 0.09 ^c^	19.70 ± 4.84 ^c^	175.9 ± 7.9 ^e^	179.8 ± 16.8 ^e^
150	0.61 ± 0.08 ^b^	20.68 ± 4.30 ^b^	168.3 ± 8.9 ^f^	163.1 ± 23.6 ^f^
180	0.80 ± 0.07 ^a^	22.89 ± 3.75 ^a^	158.1 ± 13.2 ^g^	152.4 ± 22.8 ^g^
*p*-value	0.000	0.000	0.000	0.000
Temperature × Day				
0 × 20	0.28 ± 0.01 ^i^	9.86 ± 0.09 ^l^	251.6 ± 1.9 ^a^	243.7 ± 1.9 ^a^
0 × 40	0.28 ± 0.01 ^i^	9.86 ± 0.09 ^l^	251.6 ± 1.9 ^a^	243.7 ± 1.9 ^a^
30 × 20	0.32 ± 0.02 ^h^	11.12 ± 0.58 ^i^	219.2 ± 1.1 ^b^	225.0 ± 1.4 ^b^
30 × 40	0.39 ± 0.01 ^g^	15.43 ± 0.30 ^g^	191.8 ± 0.6 ^d^	213.0 ± 1.1 ^c^
60 × 20	0.38 ± 0.02 ^g^	11.89 ± 0.69 ^i^	210.5 ± 2.2 ^c^	197.6 ± 1.3 ^d^
60 × 40	0.45 ± 0.03 ^f^	18.25 ± 0.28 ^e^	184.9 ± 1.7 ^e^	177.3 ± 1.7 ^f^
90 × 20	0.41 ± 0.02 ^g^	14.60 ± 0.15 ^h^	191.0 ± 1.4 ^d^	196.5 ± 1.2 ^d^
90 × 40	0.52 ± 0.02 ^e^	22.52 ± 0.03 ^c^	179.0 ± 1.4 ^f^	171.7 ± 1.9 ^g^
120 × 20	0.46 ± 0.02 ^f^	15.52 ± 0.24 ^g^	182.6 ± 0.2 ^e^	194.4 ± 1.2 ^d^
120 × 40	0.61 ± 0.04 ^d^	23.88 ± 0.62 ^b^	169.1 ± 1.6 ^g^	165.3 ± 1.3 ^h^
150 × 20	0.54 ± 0.03 ^e^	16.96 ± 0.21 ^f^	175.9 ± 1.4 ^f^	183.5 ± 1.9 ^e^
150 × 40	0.68 ± 0.02 ^c^	24.40 ± 0.16 ^b^	160.6 ± 1.5 ^h^	142.7 ± 1.9 ^i^
180 × 20	0.75 ± 0.01 ^b^	19.65 ± 0.21 ^d^	169.6 ± 0.8 ^g^	172.1 ± 1.4 ^g^
180 × 40	0.86 ± 0.02 ^a^	26.12 ± 0.54 ^a^	146.7 ± 0.5 ^i^	132.7 ± 1.2 ^l^
*p*-value	0.001	0.000	0.000	0.000

Each value represents the mean ± SD. Different superscripts within the same column indicate significant differences as assessed by a two-way ANOVA, followed by Fisher’s Least Significant Difference (LSD) test applied as a post hoc analysis to determine pairwise differences between means.

## Data Availability

The original contributions presented in the study are included in the article, further inquiries can be directed to the corresponding author.

## References

[1] Delshadi R., Bahrami A., Tafti A.G., Barba F.J., Williams L.L. (2020). Micro and Nano-Encapsulation of Vegetable and Essential Oils to Develop Functional Food Products with Improved Nutritional Profiles. Trends Food Sci. Technol..

[2] Bhuva S.S., Dhamsaniya N.K. (2023). Encapsulation of Vegetable Oils for Enhancing Oxidative Stability of PUFA. Bhuva Dhamsaniya Biol. Forum-Int. J..

[3] Lammari N., Louaer O., Meniai A.H., Fessi H., Elaissari A. (2021). Plant Oils: From Chemical Composition to Encapsulated Form Use. Int. J. Pharm..

[4] Shahbaz M., Sacanella E., Tahiri I., Casas R., Preedy V.R., Watson R.R. (2021). Chapter 17—Mediterranean Diet and Role of Olive Oil. Olives and Olive Oil in Health and Disease Prevention.

[5] Conte P., Squeo G., Difonzo G., Caponio F., Fadda C., Del Caro A., Urgeghe P.P., Montanari L., Montinaro A., Piga A. (2019). Change in Quality during Ripening of Olive Fruits and Related Oils Extracted from Three Minor Autochthonous Sardinian Cultivars. Emir. J. Food Agric..

[6] Patricia Blanch G., Flores G., Gómez-Jiménez M.C., Luisa Ruiz del Castillo M. (2018). Effect of the Treatment of the Olive Tree (*Olea Europaea* L.) on the Phenolic Content and Antioxidant Properties in Olive Fruits. J. Food Nutr. Res..

[7] Cerretani L., Bendini A., Del Caro A., Piga A., Vacca V., Caboni M.F., Toschi T.G. (2006). Preliminary Characterisation of Virgin Olive Oils Obtained from Different Cultivars in Sardinia. Eur. Food Res. Technol..

[8] Kiritsakis A., Kanavouras A., Kiritsakis K. (2002). Chemical Analysis, Quality Control and Packaging Issues of Olive Oil. Eur. J. Lipid Sci. Technol..

[9] Nasopoulou C., Karantonis H.C., Detopoulou M., Demopoulos C.A., Zabetakis I. (2014). Exploiting the Anti-Inflammatory Properties of Olive (*Olea Europaea*) in the Sustainable Production of Functional Food and Neutraceuticals. Phytochem. Rev..

[10] Jimenez-Lopez C., Gallardo-Gomez M., Simal-Gandara J., Carpena M., Lorenzo J.M., Lourenço-Lopes C., Barba F.J., Prieto M.A. (2020). Bioactive Compounds and Quality of Extra Virgin Olive Oil. Foods.

[11] Sánchez-Villegas A., Sánchez-Tainta A. (2017). Virgin Olive Oil: A Mediterranean Diet Essential. The Prevention of Cardiovascular Disease Through the Mediterranean Diet.

[12] Barbaro B., Toietta G., Maggio R., Arciello M., Tarocchi M., Galli A., Balsano C. (2014). Effects of the Olive-Derived Polyphenol Oleuropein on Human Health. Int. J. Mol. Sci..

[13] Gorzynik-Debicka M., Przychodzen P., Cappello F., Kuban-Jankowska A., Gammazza A.M., Knap N., Wozniak M., Gorska-Ponikowska M. (2018). Potential Health Benefits of Olive Oil and Plant Polyphenols. Int. J. Mol. Sci..

[14] Gotsis E., Anagnostis P., Mariolis A., Vlachou A., Katsiki N., Karagiannis A. (2015). Health Benefits of the Mediterranean Diet: An Update of Research over the Last 5 Years. Angiology.

[15] Campestre C., Angelini G., Gasbarri C., Angerosa F. (2017). The Compounds Responsible for the Sensory Profile in Monovarietal Virgin Olive Oils. Molecules.

[16] Lin T.K., Zhong L., Santiago J.L. (2018). Anti-Inflammatory and Skin Barrier Repair Effects of Topical Application of Some Plant Oils. Int. J. Mol. Sci..

[17] Lerma-García M.J., Simó-Alfonso E.F., Chiavaro E., Bendini A., Lercker G., Cerretani L. (2009). Study of Chemical Changes Produced in Virgin Olive Oils with Different Phenolic Contents during an Accelerated Storage Treatment. J. Agric. Food Chem..

[18] Lozano-Sánchez J., Bendini A., Quirantes-Piné R., Cerretani L., Segura-Carretero A., Fernández-Gutiérrez A. (2013). Monitoring the Bioactive Compounds Status of Extra-Virgin Olive Oil and Storage by-Products over the Shelf Life. Food Control.

[19] Polavarapu S., Oliver C.M., Ajlouni S., Augustin M.A. (2011). Physicochemical Characterisation and Oxidative Stability of Fish Oil and Fish Oil-Extra Virgin Olive Oil Microencapsulated by Sugar Beet Pectin. Food Chem..

[20] Deiana P., Santona M., Dettori S., Culeddu N., Dore A., Molinu M.G. (2019). Multivariate Approach to Assess the Chemical Composition of Italian Virgin Olive Oils as a Function of Variety and Harvest Period. Food Chem..

[21] Deiana P., Motroni A., Filigheddu M.R., Dettori S., Nieddu G., Mercenaro L., Alfei B., Culeddu N., Santona M. (2023). Effect of Pedoclimatic Variables on Analytical and Organoleptic Characteristics in Olive Fruit and Virgin Olive Oil. Eur. J. Agron..

[22] Calvo P., Hernández T., Lozano M., González-Gómez D. (2010). Microencapsulation of Extra-Virgin Olive Oil by Spray-Drying: Influence of Wall Material and Olive Quality. Eur. J. Lipid Sci. Technol..

[23] Chan L.W., Lim L.T., Heng P.W.S. (2000). Microencapsulation of Oils Using Sodium Alginate. J. Microencapsul..

[24] Sriamornsak P., Thirawong N., Puttipipatkhachorn S. (2004). Morphology and Buoyancy of Oil-Entrapped Calcium Pectinate Gel Beads. AAPS J..

[25] Velázquez-Gutiérrez S.K., Alpizar-Reyes E., Guadarrama-Lezama A.Y., Báez-González J.G., Alvarez-Ramírez J., Pérez-Alonso C. (2021). Influence of the Wall Material on the Moisture Sorption Properties and Conditions of Stability of Sesame Oil Hydrogel Beads by Ionic Gelation. LWT.

[26] Patel A.R. (2018). Structuring Edible Oils with Hydrocolloids: Where Do We Stand?. Food Biophys..

[27] Turasan H., Sahin S., Sumnu G. (2015). Encapsulation of Rosemary Essential Oil. LWT.

[28] Vergallo C. (2020). Nutraceutical Vegetable Oil Nanoformulations for Prevention and Management of Diseases. Nanomaterials.

[29] Kurek M., Descours E., Poldan P., Julou A., Pitois A., Klepac D., Vallet N., Galić K. (2024). Possibility of Storing Olive Oil in Antioxidant Biobased Pouches Made of Chitosan and Gelatin. Food Hydrocoll..

[30] Gutiérrez-luna K., Ansorena D., Astiasarán I. (2022). Use of Hydrocolloids and Vegetable Oils for the Formulation of a Butter Replacer: Optimization and Oxidative Stability. LWT.

[31] Rezagholizade-shirvan A., Soltani M., Shokri S., Radfar R., Arab M., Shamloo E. (2024). Bioactive Compound Encapsulation: Characteristics, Applications in Food Systems, and Implications for Human Health. Food Chem. X.

[32] Gao Y., Liu R., Liang H. (2024). Food Hydrocolloids: Structure, Properties, and Applications. Foods.

[33] Saha D., Bhattacharya S. (2010). Hydrocolloids as Thickening and Gelling Agents in Food: A Critical Review. J. Food Sci. Technol..

[34] Leroux J., Langendorff V., Schick G., Vaishnav V., Mazoyer J. (2003). Emulsion Stabilizing Properties of Pectin. Food Hydrocoll..

[35] Rolin C.P., Whistler R., Bemiller J.N. (1993). Industrial Gums.

[36] Lazaridou A., Biliaderis C.G. (2020). Edible Films and Coatings with Pectin.

[37] Bhatia S., Al-Harrasi A., Shah Y.A., Saif Alrasbi A.N., Jawad M., Koca E., Aydemir L.Y., Alamoudi J.A., Almoshari Y., Mohan S. (2024). Structural, Mechanical, Barrier and Antioxidant Properties of Pectin and Xanthan Gum Edible Films Loaded with Grapefruit Essential Oil. Heliyon.

[38] Caporaso N., Genovese A., Burke R., Barry-Ryan C., Sacchi R. (2016). Effect of Olive Mill Wastewater Phenolic Extract, Whey Protein Isolate and Xanthan Gum on the Behaviour of Olive O/W Emulsions Using Response Surface Methodology. Food Hydrocoll..

[39] Lagoueyte N., Paquin P. (1998). Effects of Microfluidization on the Functional Properties of Xanthan Gum. Food Hydrocoll..

[40] Sun C., Gunasekaran S., Richards M.P. (2007). Effect of Xanthan Gum on Physicochemical Properties of Whey Protein Isolate Stabilized Oil-in-Water Emulsions. Food Hydrocoll..

[41] de Morais Lima M., Bianchini D., Guerra Dias A., da Rosa Zavareze E., Prentice C., da Silveira Moreira A. (2017). Biodegradable Films Based on Chitosan, Xanthan Gum, and Fish Protein Hydrolysate. J. Appl. Polym. Sci..

[42] Bascuas S., Hernando I., Moraga G., Quiles A. (2020). Structure and Stability of Edible Oleogels Prepared with Different Unsaturated Oils and Hydrocolloids. Int. J. Food Sci. Technol..

[43] Bouyer E., Mekhloufi G., Huang N., Rosilio V., Agnely F. (2013). β-Lactoglobulin, Gum Arabic, and Xanthan Gum for Emulsifying Sweet Almond Oil: Formulation and Stabilization Mechanisms of Pharmaceutical Emulsions. Colloids Surf. A Physicochem. Eng. Asp..

[44] Cortes H., Caballero-Florán I.H., Mendoza-Muñoz N., Escutia-Guadarrama L., Figueroa-González G., Reyes-Hernández O.D., González-Del Carmen M., Varela-Cardoso M., González-Torres M., Florán B. (2020). Xanthan Gum in Drug Release. Cell. Mol. Biol..

[45] Paba A., Chessa L., Cabizza R., Daga E., Urgeghe P.P., Testa M.C., Comunian R. (2019). Zoom on Starter Lactic Acid Bacteria Development into Oxytetracycline Spiked Ovine Milk during the Early Acidification Phase. Int. Dairy J..

[46] Petri D.F.S. (2015). Xanthan Gum: A Versatile Biopolymer for Biomedical and Technological Applications. J. Appl. Polym. Sci..

[47] Sharma S., Rao T.V.R. (2015). Xanthan Gum Based Edible Coating Enriched with Cinnamic Acid Prevents Browning and Extends the Shelf-Life of Fresh-Cut Pears. LWT.

[48] Barbosa de Almeida C., Catelam K.T., Lopes Cornélio M., Lopes Filho J.F. (2010). Morphological and Structural Characteristics of Zein Biofilms with Added Xanthan Gum. Food Technol. Biotechnol..

[49] Dahdah P., Cabizza R., Farbo M.G., Fadda C., Mara A., Hassoun G., Piga A. (2024). Improving the Rheological Properties of Dough Obtained by Partial Substitution of Wheat Flour with Freeze-Dried Olive Pomace. Foods.

[50] Dahdah P., Cabizza R., Farbo M.G., Fadda C., Del Caro A., Montanari L., Hassoun G., Piga A. (2024). Effect of Partial Substitution of Wheat Flour with Freeze-Dried Olive Pomace on the Technological, Nutritional, and Sensory Properties of Bread. Front. Sustain. Food Syst..

[51] (1991). Commission Regulation (EEC) No 2568/91 of 11 July 1991 on the Characteristics of Olive Oil and Olive-Residue Oil and on the Relevant Methods of Analysis. Off. J. Eur. Communities.

[52] Singleton V.L., Rossi J.A. (1965). Colorimetry of Total Phenolics with Phosphomolybdic-Phosphotungstic Acid Reagents. Am. J. Enol. Vitic..

[53] Gimeno E., Castellote A.I., Lamuela-Raventós R.M., de la Torre M.C., López-Sabater M.C. (2000). Rapid Determination of Vitamin E in Vegetable Oils by Reversed-Phase High-Performance Liquid Chromatography. J. Chromatogr. A.

[54] Patel A.R., Cludts N., Bin Sintang M.D., Lesaffer A., Dewettinck K. (2014). Edible Oleogels Based on Water Soluble Food Polymers: Preparation, Characterization and Potential Application. Food Funct..

[55] Patel A.R., Cludts N., Bin Sintang M.D., Lewille B., Lesaffer A., Dewettinck K. (2014). Polysaccharide-Based Oleogels Prepared with an Emulsion-Templated Approach. ChemPhysChem.

[56] Giri T.K., Choudhary C., Alexander A., Ajazuddin A., Badwaik H., Tripathy M., Tripathi D.K. (2013). Sustained Release of Diltiazem Hydrochloride from Cross-Linked Biodegradable IPN Hydrogel Beads of Pectin and Modified Xanthan Gum. Indian J. Pharm. Sci..

[57] Garrido J.I., Lozano J.E., Genovese D.B. (2015). Effect of Formulation Variables on Rheology, Texture, Colour, and Acceptability of Apple Jelly: Modelling and Optimization. LWT.

[58] Goulao L.F., Oliveira C.M. (2008). Cell Wall Modifications during Fruit Ripening: When a Fruit Is Not the Fruit. Trends Food Sci. Technol..

[59] Fraeye I., De Roeck A., Duvetter T., Verlent I., Hendrickx M., Van Loey A. (2007). Influence of Pectin Properties and Processing Conditions on Thermal Pectin Degradation. Food Chem..

[60] Kalua C.M., Allen M.S., Bedgood D.R., Bishop A.G., Prenzler P.D., Robards K. (2007). Olive Oil Volatile Compounds, Flavour Development and Quality: A Critical Review. Food Chem..

[61] Morelló J.R., Motilva M.J., Tovar M.J., Romero M.P. (2004). Changes in Commercial Virgin Olive Oil (Cv Arbequina) during Storage, with Special Emphasis on the Phenolic Fraction. Food Chem..

[62] Chabni A., Bañares C., Torres C.F. (2024). Study of the Oxidative Stability via Oxitest and Rancimat of Phenolic-Rich Olive Oils Obtained by a Sequential Process of Dehydration, Expeller and Supercritical CO_2_ Extractions. Front. Nutr..

[63] Migliorini M., Cherubini C., Cecchi L., Zanoni B. (2013). Degradazione Dei Composti Fenolici Durante La Conservazione Dell’olio Extra Vergine Di Oliva. Riv. Ital. delle Sostanze Grasse.

[64] Krichene D., Salvador M.D., Fregapane G. (2015). Stability of Virgin Olive Oil Phenolic Compounds during Long-Term Storage (18 Months) at Temperatures of 5–50 °C. J. Agric. Food Chem..

[65] Castillo-Luna A., Criado-Navarro I., Ledesma-Escobar C.A., López-Bascón M.A., Priego-Capote F. (2021). The Decrease in the Health Benefits of Extra Virgin Olive Oil during Storage Is Conditioned by the Initial Phenolic Profile. Food Chem..

[66] Mancebo-Campos V., Salvador M.D., Fregapane G. (2022). Modelling Virgin Olive Oil Potential Shelf-Life from Antioxidants and Lipid Oxidation Progress. Antioxidants.

[67] Caipo L., Sandoval A., Sepúlveda B., Fuentes E., Valenzuela R., Metherel A.H., Romero N. (2021). Effect of Storage Conditions on the Quality of Arbequina Extra Virgin Olive Oil and the Impact on the Composition of Flavor-Related Compounds (Phenols and Volatiles). Foods.

[68] Verleyen T., Kamal-Eldin A., Dobarganes C., Verhe R., Dewettinck K., Huyghebaert A. (2001). Modeling of α-Tocopherol Loss and Oxidation Products Formed during Thermoxidation in Triolein and Tripalmitin Mixtures. Lipids.

